# Supplementary material for: The HP1 box of KAP1 organizes HP1α for silencing of endogenous retroviral elements in embryonic stem cells
Source: Nat Commun. 2025 May 31;16:5066. doi: 10.1038/s41467-025-60279-2 (PMC12126490; doi:10.1038/s41467-025-60279-2)
Supplement: Supplementary file 1 — Supplementary Information [file 41467_2025_60279_MOESM1_ESM.pdf]

Supplementary Information

The HP1 box of KAP1 organizes HP1α for silencing of endogenous retroviral elements in embryonic stem cells

Nitika Gaurav, Ryan O'Hara, et al.

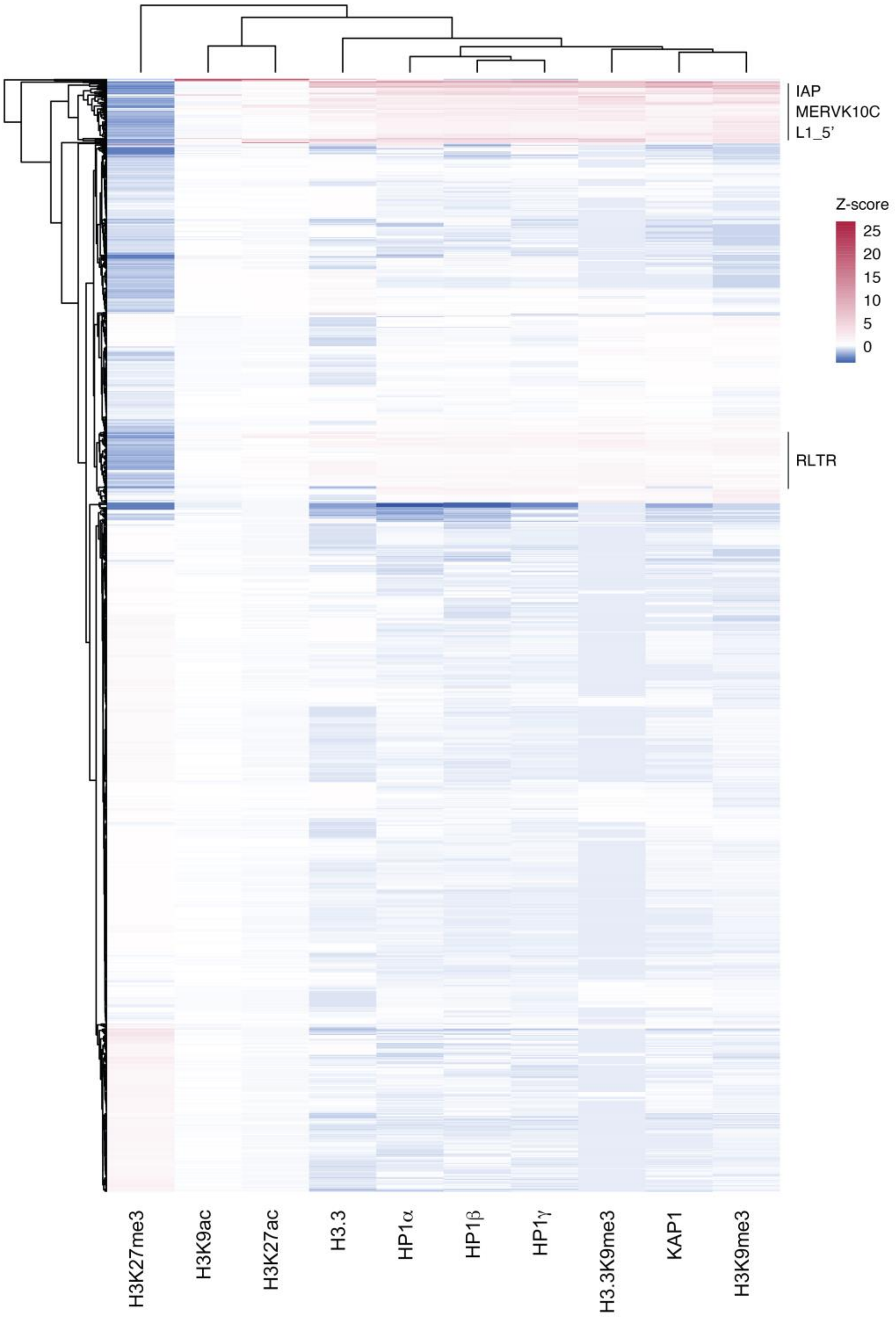

Supplementary Figure 1. KAP1 and HP1 are enriched at endogenous retroelements in mouse embryonic stem cells. ChIP-seq enrichment of heterochromatic histone modifications and factors, including HP1 proteins, mapped to the repetitive genome. Data are represented in a hierarchically (Spearman rank) clustered heatmap of z-score fold enrichment (red) or depletion (blue) over a matched input. Related to Figure 1.

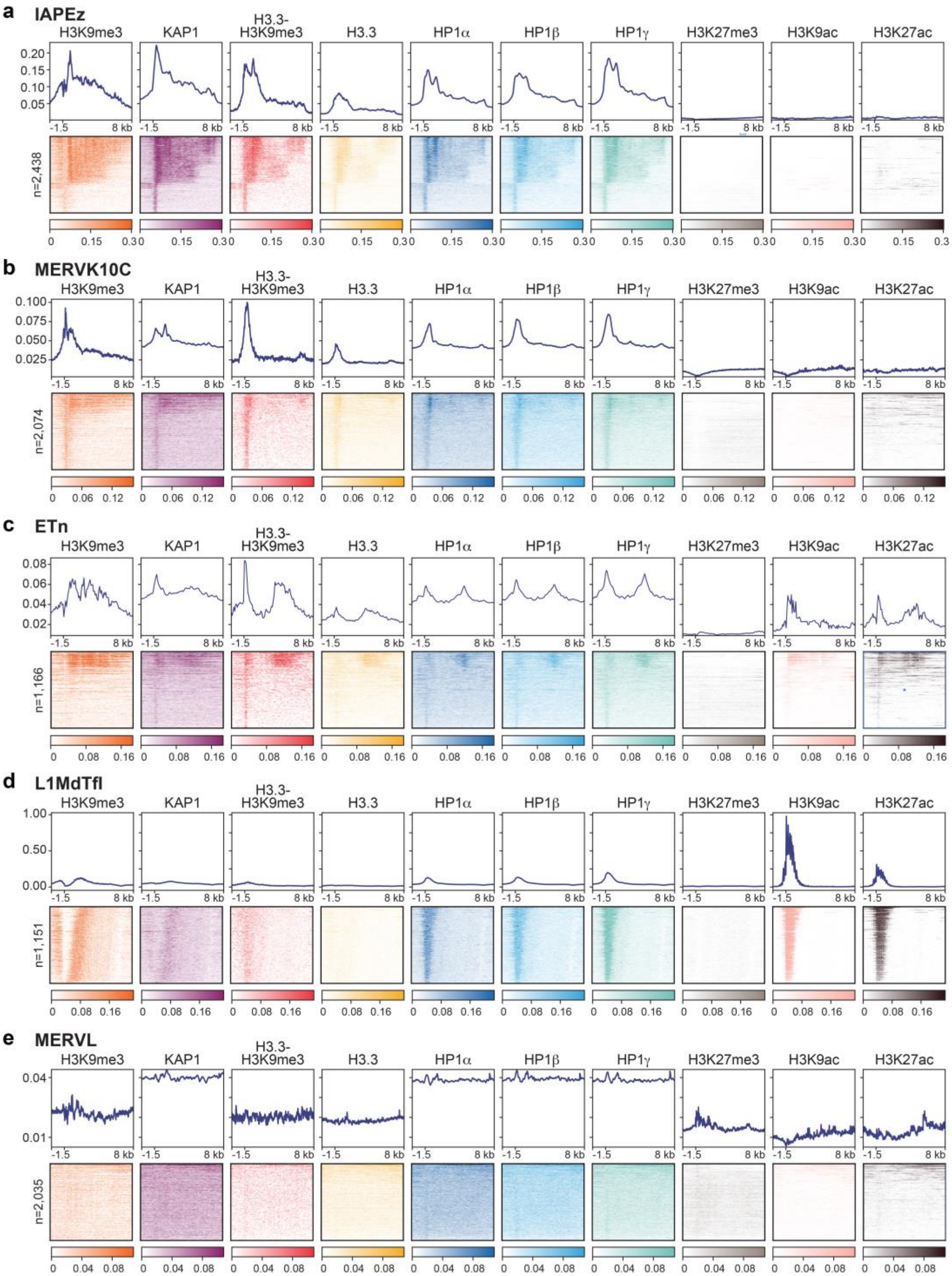

Supplementary Figure 2. KAP1 and HP1 co-occupy ERVs in mouse ESCs. (a-e) Average profiles (top) and heatmaps (bottom) of histone modification and chromatin protein enrichment at (a) IAPEz (n = 2,438), (b) MERVK10C (n = 2,074), (c) ETn (n=1,116), (d) L1MdTfI (n=1,151), and (e) MERVL (n=2,035) in ESCs. Data are centered on the LTR with 1.5 kb upstream and 8 kb downstream of the LTR displayed for each analysis. Related to Figure 1.

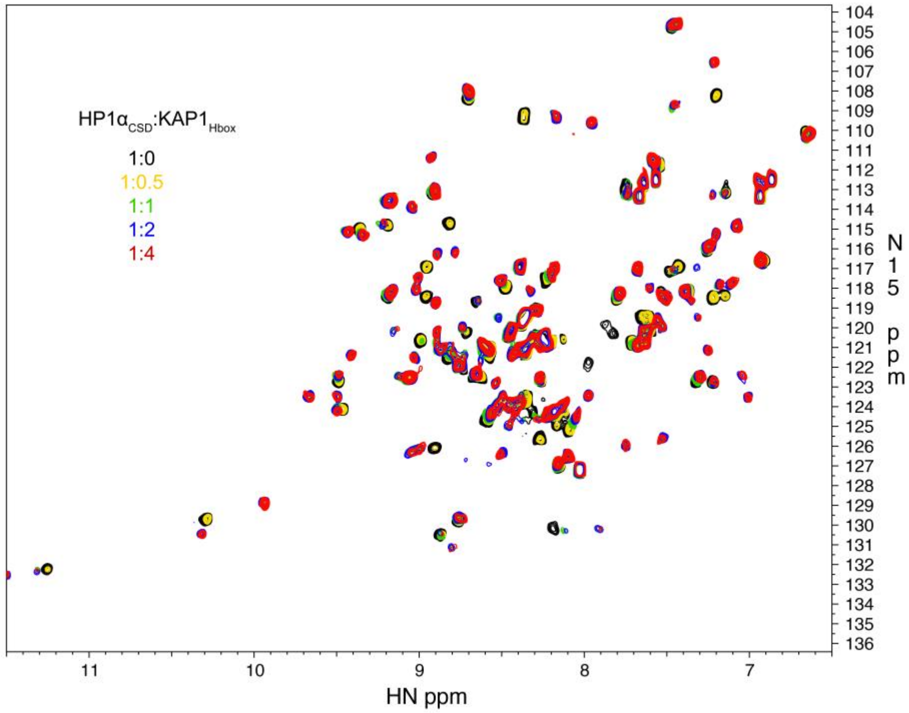

Supplementary Figure 3. Overlayed ^{1}H,^{15}N HSQC spectra of ^{15}N-labeled HP1α$_{CSD}$ recorded in the presence of increasing amount of KAP1$_{Hbox}$ peptide. Spectra are color coded according to the protein:peptide molar ratio. Related to Figure 3.

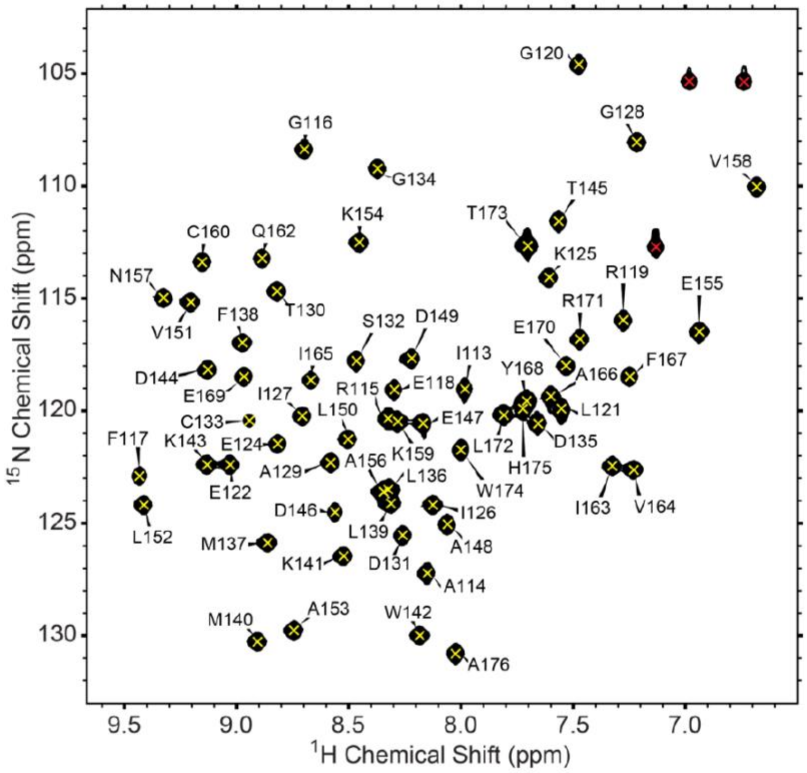

Supplementary Figure 4. ^{1}H,^{15}N HSQC NMR spectrum of HP1α$_{CSD'}$ (aa 112-176 of HP1α). Assigned crosspeaks are labeled. The crosspeaks marked with a red 'x' are from the amide side chains. Related to Figure 3.

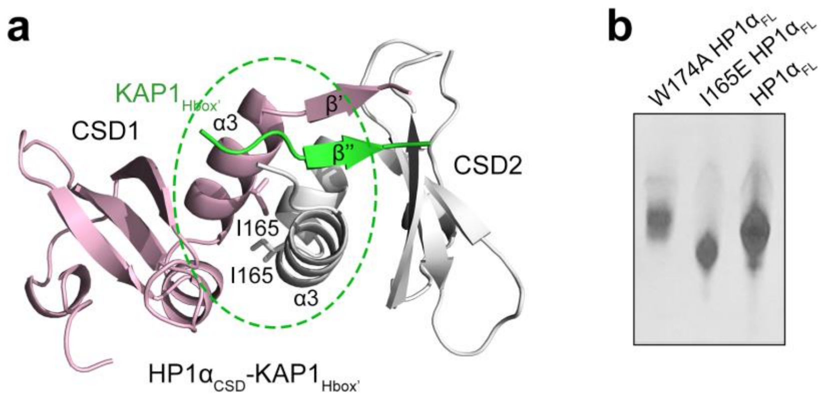

Supplementary Figure 5. (a) Ribbon diagram of the HP1α_{CSD}-KAP1$_{Hbox'}$ complex, with I165 in α3, which is essential for the HP1α_{CSD} dimerization, shown as stick and labeled. (b) Native-PAGE gel showing migration of full-length wild type and mutated full length HP1α. Related to Figure 3.

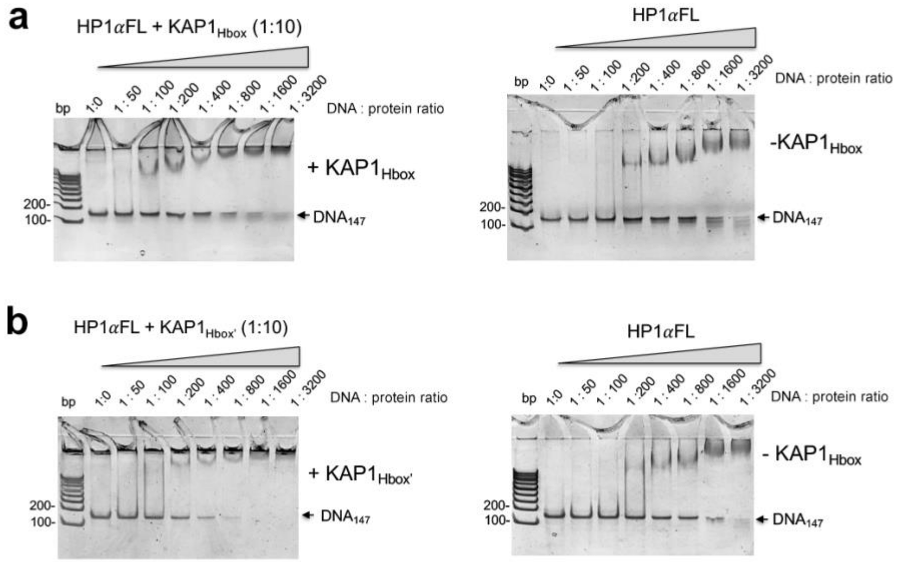

Supplementary Figure 6. (a) EMSA of 147 bp Widom 601 DNA in the presence of increasing amounts of full length HP1α with and without tenfold molar excess of $KAP1_{Hbox}$. DNA:protein ratio is shown above the gel images. Repeat of EMSA experiments shown in Fig. 4a. (b) EMSA of 147 bp 601 DNA in the presence of increasing amounts of full length HP1α with and without tenfold molar excess of $KAP1_{Hbox'}$. DNA:protein ratio is shown above the gel images. Related to Figure 4. Source data are provided as a Source Data file.

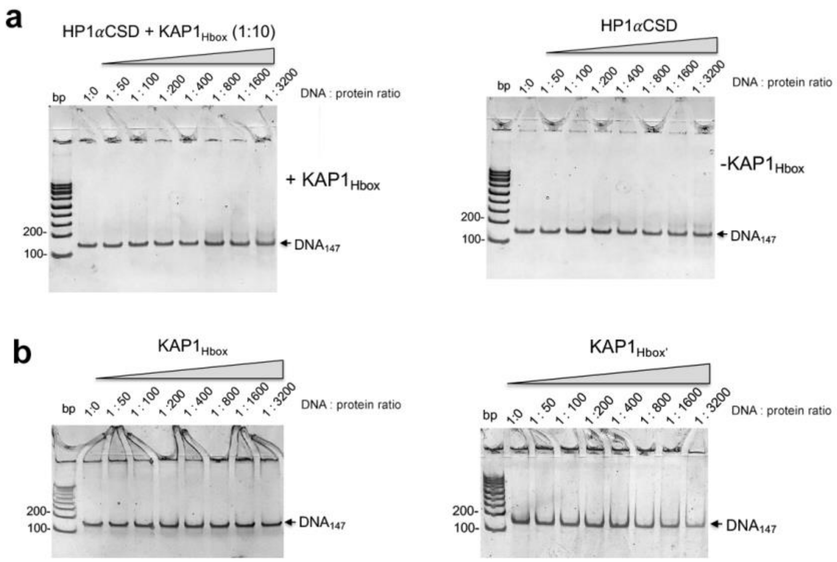

Supplementary Figure 7. (a) EMSA of 147 bp Widom 601 DNA in the presence of increasing amounts of HP1α$_{CSD}$ with and without tenfold molar excess of KAP1$_{Hbox}$. DNA:protein ratio is shown above the gel images. (b) EMSA of 147 bp 601 DNA in the presence of increasing amounts of KAP1$_{Hbox}$ or KAP1$_{Hbox'}$. DNA:protein ratio is shown above the gel images. Related to Figure 4. Source data are provided as a Source Data file.

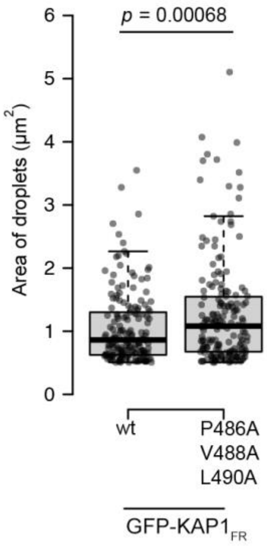

Supplementary Figure 8. The box plot displays the size of HP1α phase-separated droplets. Center lines show the medians; box limits indicate the 25th and 75th percentiles as determined by R software; whiskers extend 1.5 times the interquartile range from the 25th and 75th percentiles. n = 169 (wild type GFP-KAP1$_{RF}$), 175 (GFP-KAP1$_{RF}$ P486A/V488A/L490A mutant). A student-t test, p-value is indicated. Related to Figure 4.

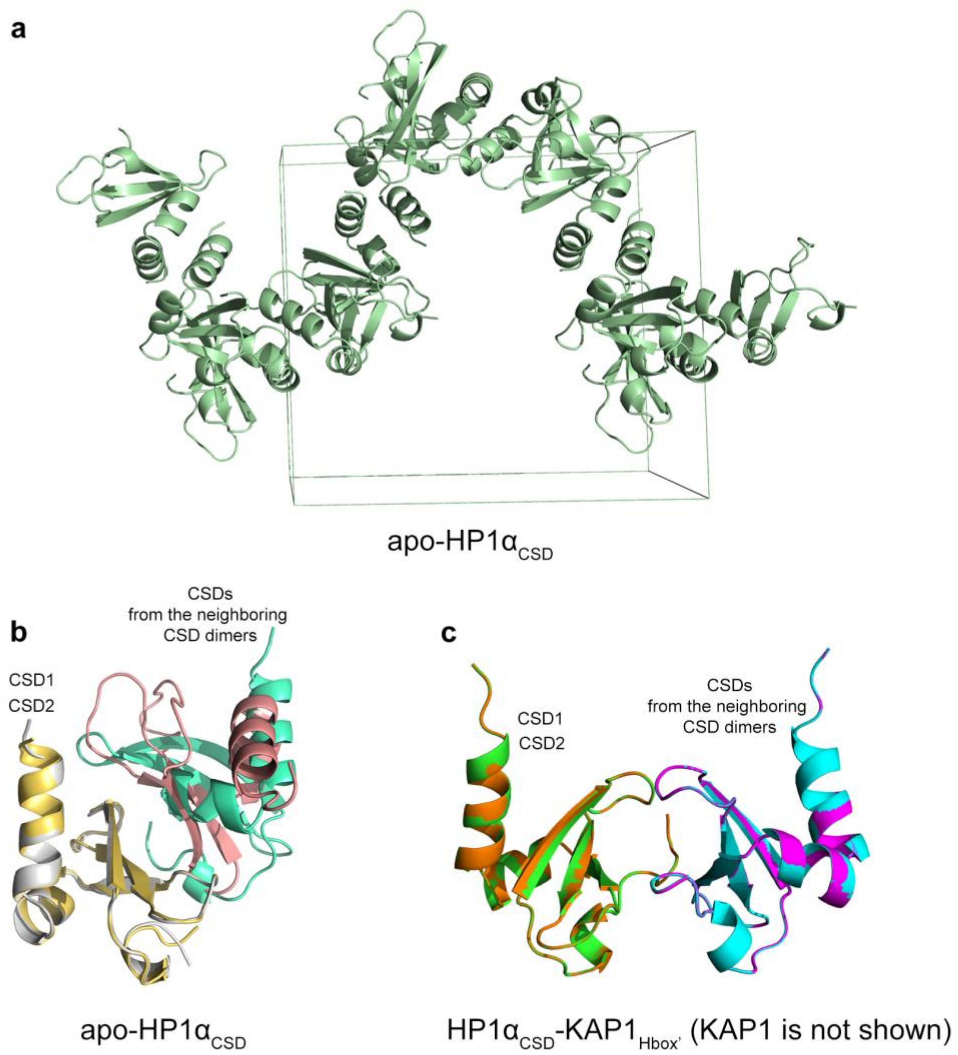

Supplementary Figure 9. (a) Spatial arrangement of CSDs in the unit cell of apo-state of HP1α. (b) In the apo-state of HP1α, the CSD1 and CSD2 protomers are superimposed, however CSDs from the neighboring CSD dimers cannot be superimposed. (c) In contrast, in the complex with $KAP1_{Hbox'}$ peptide, CSD1 and CSD2 protomers are superimposed, as well as CSDs from the neighboring CSD dimers are superimposed (RMSDs = 0 Å). Related to Figures 5 and 6.

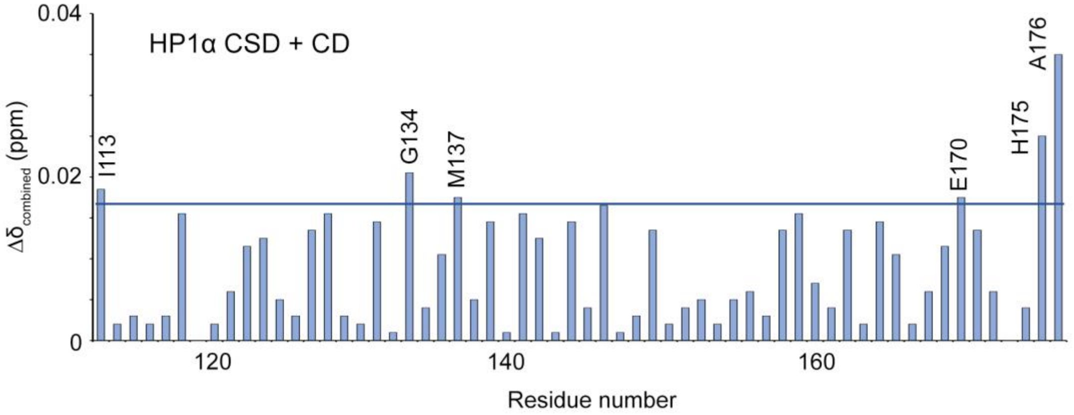

Supplementary Figure 10. Bar plot of resonance changes in ^{1}H,^{15}N HSQC spectra of ^{15}N-labeled HP1α$_{CSD'}$ induced by the 18-fold molar excess of unlabeled HP1α$_{CD}$, as control. Related to Figure 5.

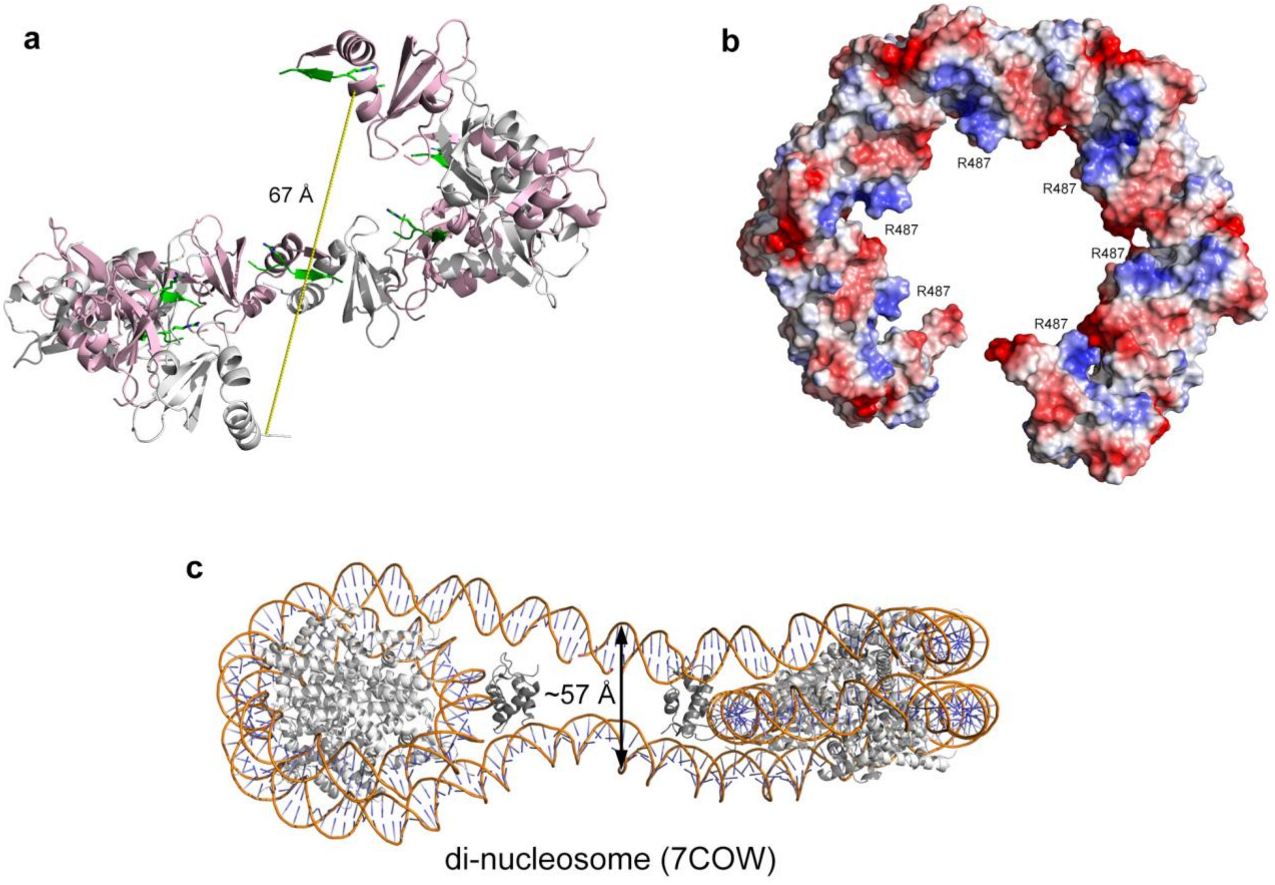

Supplementary Figure 11. Structural organization of the symmetrical multimer of the HP1α_{CSD}-KAP1$_{Hbox}$ complex: (a) ribbon diagram and (b) electrostatic surface potential ranging from positive;blue (+100 kT/e) to negative;red (−100 kT/e). (c) The structure of the dinucleosome in complex with H1, PDB ID 7COW. Related to Figure 6.

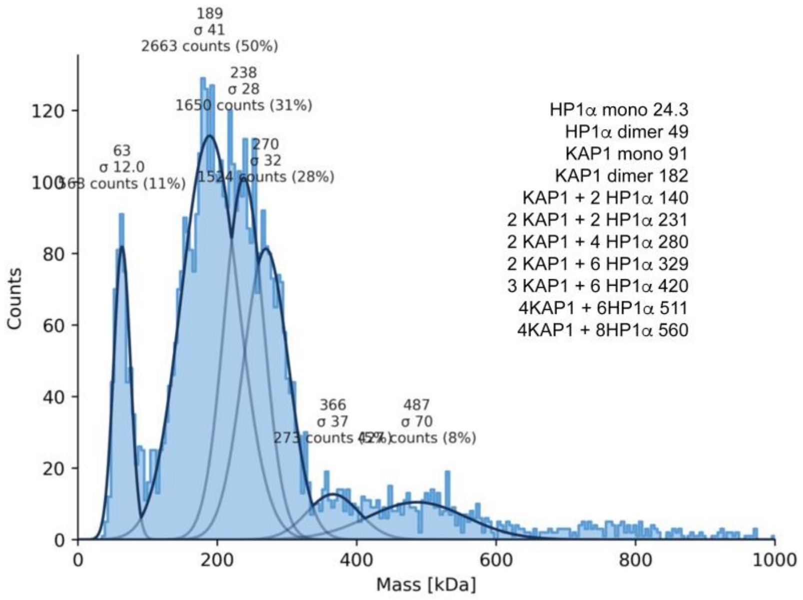

Supplementary Figure 12. Molecular mass distribution histogram of the 2:1 mixture of full length HP1α and full length KAP1 in mass photometry assay. Maxima of the fits are labeled in kDa. Total counts and % of total counts for each peak are also shown. Theoretical molecular masses of indicated proteins and complexes (kDa) are shown on the right. Related to Figure 6.

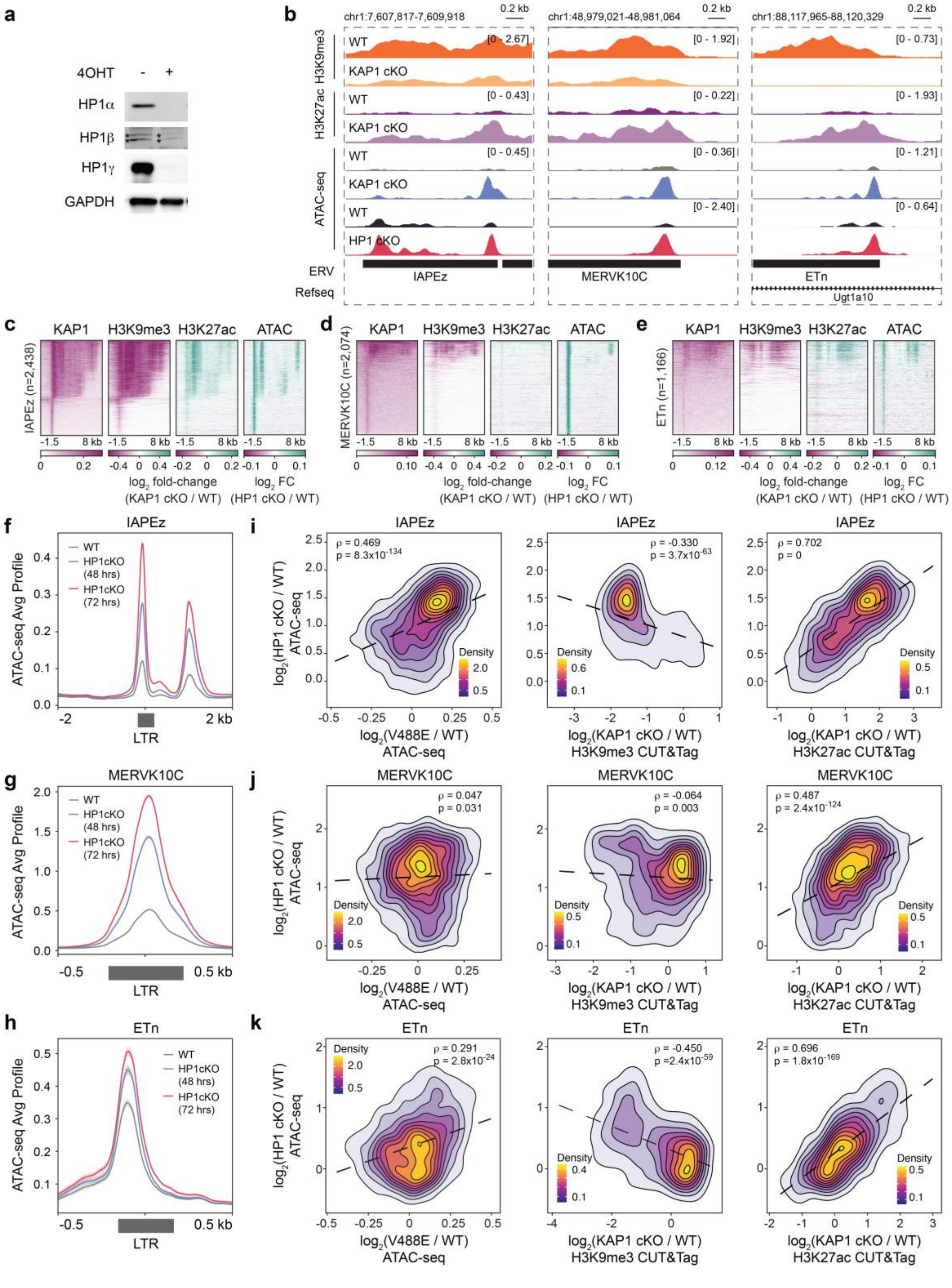

Supplementary Figure 13. HP1 contributes to inaccessible chromatin at ERVs in ESCs. (a) Immunoblot of whole cell lysates from *Cbx1-/-;Cbx3fl/fl;Cbx5fl/fl;Cre-ERT2* ESCs [Ostapcuk, 2018]. ESCs were treated with either EtOH or 2 μM 4-Hydroxytamoxifen for 72 hrs. (b) Genome browser

representations of H3K9me3 and H3K27ac CUT&Tag in WT and KAP1 cKO ESCs as well as ATAC-seq in WT and KAP1 cKO ESCs and WT and HP1 cKO ESCs at representative ERVs. The y axis represents read density in counts per million (CPM) mapped reads for an experiment performed in duplicate or triplicate. (c-e) Heatmaps of KAP1 enrichment, the relative H3K9me3 and H3K27ac CUT&Tag enrichment difference between WT and KAP1 cKO, and the relative ATAC-seq enrichment difference between HP1 cKO ESCs and WT ESCs at (c) IAPEz (n=2,438), (d) MERVK10C (n=2,074), and (e) ETn (n=1,1166) ERVs. (f-h) ATAC-seq average profiles in WT and HP1 cKO ESCs treated with 2 μM 4-OHT for 48 or 72 hrs at LTRs of (f) IAPEz (n=2,438, p=0.0004), (g) MERVK10C (n = 2,074, $p=7.94x10^{-12}$) and (h) ETn (n=1,166, p=0.0003)). LTRs were either orphan or adjacent to full-length elements as defined in the methods. (i-k) Correlation plots between differential ATAC-seq signal in WT and HP1 cKO ESCs compared to differential ATAC-seq signal in KAP1 cKO ESCs expressing either WT or KAP1 V488E (left), differential H3K9me3 CUT&Tag in KAP1 cKO compared to WT ESCs (middle), and differential H3K27ac CUT&Tag in KAP1 cKO compared to WT ESCs (left) at (i) IAPEz (n=2,438), (j) MERVK10C (n=2,074), and (k) ETn (n=1,166). Significance determined by Wilcoxon rank sum with BH correction. Related to Figure 7.

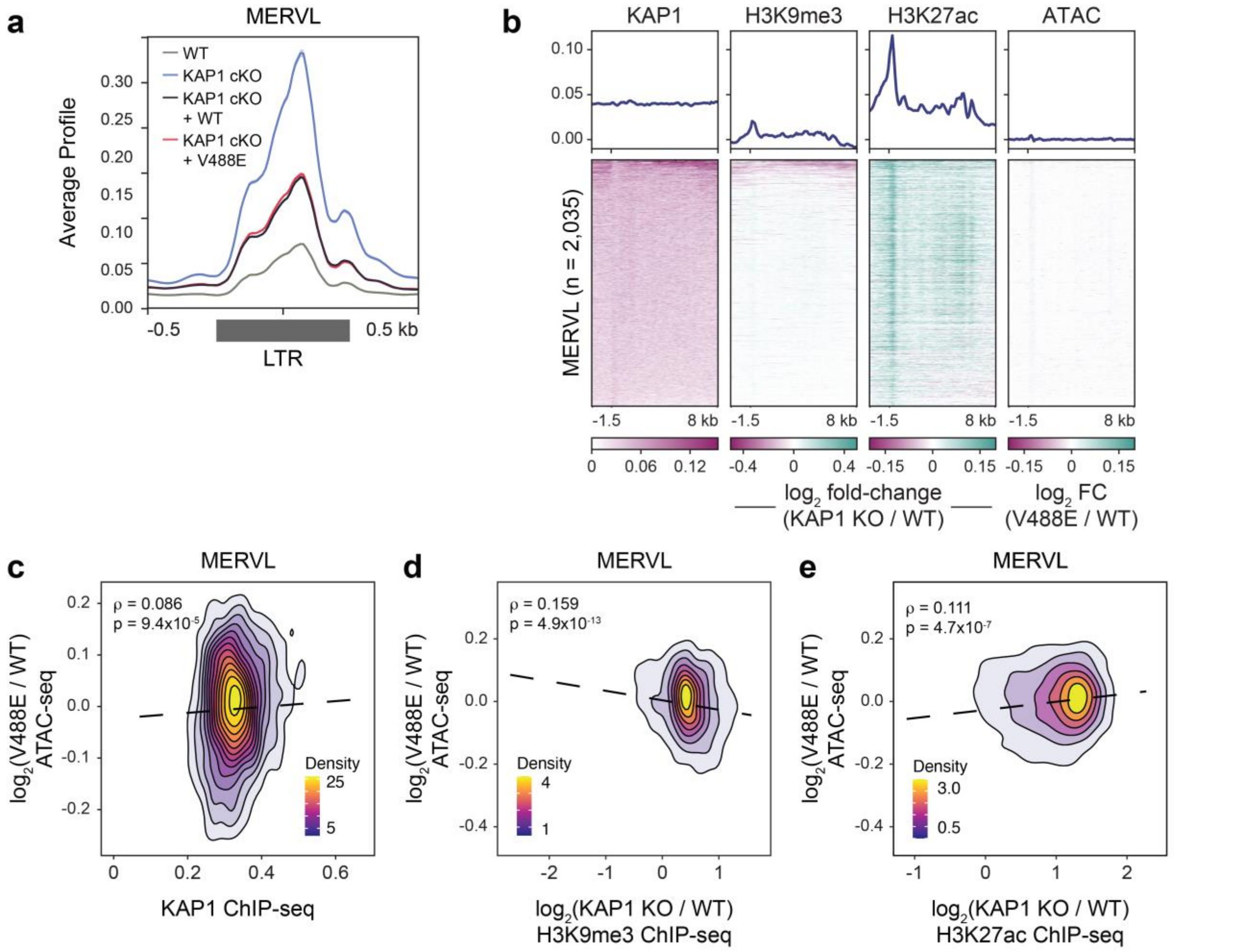

Supplementary Figure 14. MERVL accessibility is independent of KAP1-HP1 interaction.
(a) ATAC-seq average profiles in WT and KAP1 cKO ESCs and KAP1 cKO ESCs expressing exogenous KAP1 or a KAP1 V488E mutant at LTRs of MERVL (n=2,035, p=0.06). LTRs were either orphan or adjacent to full-length elements as defined in the methods. Significance reported for KAP1 vs KAP1 V488E addback in KAP1 cKO as determined by Wilcoxon rank sum with BH correction. (b) Average profiles (top) and heatmaps (bottom) of KAP1 enrichment, the relative H3K9me3 and H3K27ac enrichment difference between WT and KAP1 cKO, and the relative ATAC-seq enrichment difference between KAP1 cKO ESCs expressing either WT or KAP1 V488E at MERVL (n=2,035) ERVs. (c-e) Correlation plots between differential ATAC-seq signal in KAP1 cKO ESCs expressing either WT or KAP1 V488E compared to (c) KAP1 ChIP-seq, (d) differential H3K9me3 in KAP1 cKO compared to WT ESCs, and (e) differential H3K27ac in KAP1 cKO compared to WT ESCs (left) at MERVL (n=2,035). Related to Figure 7.

Supplementary Table 1. Data collection and refinement statistics.

	HP1α CSD-apo	HP1α CSD-KAP1 Hbox'
Space group	$P2_12_12_1$	$P6_5$
Cell dimensions		
a, b, c (Å)	63.76, 66.85, 70.91	107.81, 107.81, 64.61
α, β, γ (°)	90.0, 90.0, 90.0	90.0, 90.0, 120.0
Resolution (Å)	46.14 - 2.15 (2.22 – 2.15)*	46.68-2.40 (2.49-2.40)*
R_{merge} (%)	11.8 (83.0)	7.8 (49.0)
<I/σ(I)>	11.3 (2.2)	16.5 (3.4)
$CC_{1/2}$	0.99 (0.65)	0.99 (0.94)
Completeness (%)	99.9 (99.9)	100.0 (100.0)
Unique reflections	17068 (1446)	16886 (1763)
Multiplicity	6.4 (6.2)	9.5 (6.4)
Refinement		
Resolution (Å)	46.18 – 2.15	37.87 - 2.40
No. of reflections	16249	15941
Rwork (%)	22.74	23.46
Rfree (%)	27.73	25.23
RMS Bond lengths (Å)	0.008	0.009
RMS Bond angles (°)	1.9	1.8
No. of atoms		
Protein	1882	2072
KAP1	-	94
Water	109	55
Average B ($Å^2$)		
Protein	42.01	48.51
KAP1	-	103.2
Water	41.44	37.91
Ramachandran (%)		
Favoured regions	91.9	87.0
Allowed regions	8.1	13.0
Outliers	0.0	0.0

*Values in parentheses are for highest-resolution shell.
